# Effects of Konjac Glucomannan and Inulin on the Gel Properties, Swallowing Characteristics, and In Vitro Digestion of Heat-Induced Silver Carp–Sea Cucumber Composite Gels for Dysphagia

**DOI:** 10.3390/foods15193471

**Published:** 2026-09-28

**Authors:** Jingyi Huo, Meiduo Xue, Shengjun Chen, Yangli Chen, Yumeng Wei, Lijuan Xu, Meng Li, Xinru Fan, Soottawat Benjakul, Qiancheng Zhao

**Affiliations:** 1 College of Food Science and Engineering, Dalian Ocean University, Dalian 116023, China; 2 Key Laboratory of Aquatic Product Processing, Ministry of Agriculture and Rural Affairs, South China Sea Fisheries Research Institute, Chinese Academy of Fishery Sciences, Guangzhou 510300, China; 3 International Center of Excellence in Seafood Science and Innovation, Faculty of Agro-Industry, Prince of Songkla University, Hat Yai 90110, Thailand

**Keywords:** dysphagia, texture-modified, IDDSI, adult digestion, elderly digestion

## Abstract

In this study, we used silver carp (*Hypophthalmichthys molitrix*) and sea cucumber (*Apostichopus japonicus*) as the primary protein sources and supplemented them with konjac glucomannan (KGM) or inulin (INU) at concentrations of 0%, 0.5%, 1.0%, and 1.5% to evaluate the gelling properties, textural characteristics, rheological behaviors, and in vitro digestibility of the resulting composite gels. The gastrointestinal digestion behavior, structural changes, and functional characteristics of these gels under simulated adult and elderly physiological conditions were also systematically investigated to clarify how different physiological stages influence their digestive fate. The results showed that incorporating KGM or INU modified hydrogen bonding and protein–polysaccharide interactions within the gel network, thereby regulating the structural integrity and rheological properties of the composite gels. According to the International Dysphagia Diet Standardization Initiative (IDDSI) framework, the KGM/INU-modified gels successfully passed the prescribed tests and were classified as level 4 (pureed/extremely thick) and level 6 (soft and bite-sized) foods, confirming their suitability for dysphagia management. Compared with the control group, the 0.5% KGM group showed the most desirable texture profile, with significantly lower hardness, chewiness, and adhesiveness, and higher cohesiveness (*p* < 0.05), while the 1.0% INU group exhibited the greatest enhancement in gel strength and viscoelasticity, as evidenced by the highest storage modulus (G′) and loss modulus (G″) (*p* < 0.05). Moreover, both adult and elderly in vitro digestion models showed extensive proteolysis of the composite gels, together with reduced protein aggregation, a looser microstructure in the digesta, and enhanced antioxidant capacity. Notably, the elderly model generally showed higher digestibility than the adult model, accompanied by greater release of free amino acids and antioxidant constituents (*p* < 0.05). Overall, these findings indicate that KGM/INU-modified composite gels possess promising potential as texture-modified foods, while the two in vitro physiological models provide a valuable platform for evaluating their digestion-related properties. KGM/INU-modified composite gels thus provide a promising platform for developing texture-modified, nutritionally fortified foods suitable for elderly individuals with swallowing impairments and adults who require safe, readily digestible dietary options.

## 1. Introduction

Population aging has become a major global societal challenge, with substantial implications for public health systems and nutritional care. Swallowing, or deglutition, is a highly coordinated physiological process involving complex interactions among neural, muscular, chemical, and physical reflex mechanisms, through which ingested material is transported from the oral cavity to the stomach via the esophagus [1]. Dysfunction at any stage of this complex process may result in oropharyngeal dysphagia. This condition is a prevalent health concern among older populations and is clinically characterized by impaired mastication, compromised pharyngeal swallowing, and an increased risk of choking and aspiration during food and liquid intake. These impairments can compromise nutritional status and substantially reduce the quality of life and overall well-being of affected elderly individuals [2]. With advancing age, reduced flexibility of the upper esophageal sphincter may further increase swallowing resistance, creating substantial barriers to normal dietary intake.

Patients with dysphagia commonly rely on texture-modified diets, including pureed foods and thickened liquids, to reduce the risk of aspiration and ensure adequate nutrient intake [3]. However, conventional foods developed for dysphagia management may have poor visual appeal because of their physical characteristics, which can suppress appetite and reduce dietary adherence. In contrast, gel-based food matrices have considerable potential to stimulate appetite due to their attractive appearance and adaptable texture [4]. Dietary regimens for elderly individuals also require adequate protein and essential micronutrients to maintain skeletal muscle mass and bone health. Swallowing difficulties combined with reduced appetite can, however, decrease food intake in elderly individuals, leading to inadequate energy intake and impaired nutrient consumption and absorption. Elderly people are also susceptible to oxidative stress, which exacerbates age-related degeneration. Notably, antioxidant peptides are only generated after gastrointestinal proteolysis. Hence, detecting antioxidant activity of digested hydrolysates helps evaluate the functional benefits of the composite gels for older adults with dysphagia. Bioactive peptides and other bioactive components released after gastrointestinal digestion of protein-rich foods can exert antioxidant effects and alleviate oxidative damage. This cycle can substantially compromise health status and quality of life [5].

In recent years, soft-textured foods based on protein–polysaccharide composite systems have attracted considerable research interest and are increasingly recognized as suitable dietary options for individuals with dysphagia [6]. Accumulating evidence indicates that protein–polysaccharide hybrid matrices enable effective modulation of gel-swallowing properties and textural characteristics. For example, previous studies have shown that combining whey protein isolate with tamarind seed gum produces dysphagia-friendly high-internal-phase emulsions with reduced droplet size and improved physical stability [7]. Similarly, incorporating dietary fibers, including inulin (INU) and konjac glucomannan (KGM), into golden pomfret myofibrillar protein can effectively modify the mechanical, swallowing, and sensory properties of emulsion gels while maintaining nutritional profiles comparable to those of fish proteins [6]. Furthermore, composite gels prepared from sodium alginate, Antarctic krill, and *Penaeus vannamei* surimi exhibited increased chewiness and zeta potential, along with reduced hardness and denaturation enthalpy, and were classified as level 6 foods under the International Dysphagia Diet Standardization Initiative (IDDSI) framework [8]. Combining the complementary properties of polysaccharides, particularly their favorable rheological behavior, with the nutritional functionality of proteins enables the development of diverse gel structures with high nutritional value. Such systems thus have considerable potential as transitional or texture-modified foods for individuals with dysphagia.

Silver carp (*Hypophthalmichthys molitrix*) is characterized by tender flesh and a high protein content, with its protein being rich in lysine and glutamate, thus making it a high-quality source of animal-derived protein. Because of its rapid growth rate, ease of processing, and cost-effectiveness, this species has become a major raw material for freshwater surimi production. In recent years, silver carp surimi processing has increasingly focused on developing premium, nutritionally enhanced, and health-oriented food products. Previous studies have demonstrated that silver carp surimi can form soft gels following homogenization and thermal treatment, with textural properties that meet the physical requirements of foods for individuals with dysphagia [2]. However, gels prepared solely from silver carp protein primarily provide protein and contain relatively few bioactive components. In contrast, sea cucumber (*Apostichopus japonicus*) is a marine food resource of considerable economic value. Sea cucumber body wall is rich in vitamins, minerals, and amino acids (especially arginine), as well as bioactive substances such as peptides, saponins, cerebrosides, and polysaccharides, which exhibit various physiological activities, including anti-inflammatory, antitumor, and immunoenhancing effects [9]. During the processing of ready-to-eat sea cucumbers, there are some individuals with smaller size or damaged shape, which diminishes their commercial value despite the fact that their nutritional integrity remains largely unimpaired and their edible potential is preserved. If these low-value ready-to-eat sea cucumbers can be further processed, their utilization rate as raw materials will be effectively increased and their economic value enhanced. Nevertheless, the inherently tough texture of sea cucumber makes it unsuitable for direct consumption by individuals with swallowing impairments, highlighting the need to develop novel sea cucumber-based composite gel products tailored to this population. Therefore, given the benefits in amino acid complementation, texture modification, and utilization of low-value raw materials, developing a silver carp-sea cucumber complex gel is highly worthwhile.

Thus, in this study, we developed thermally induced composite gels containing sea cucumber (*A. japonicus*) and silver carp (*H. molitrix*) with varying concentrations of KGM and INU to systematically investigate their potential as foods for individuals with dysphagia. A multifaceted characterization approach was used, including Fourier transform infrared (FTIR) spectroscopy, quantification of intermolecular forces, texture profile analysis (TPA), rheological measurements, and evaluation using the IDDSI framework. In parallel, adult and elderly static in vitro digestion models were employed to assess the digestibility and amino acid profiles of the resulting hydrolysates. The microstructural imaging, protein conformational characterization, and antioxidant activity assay were conducted to identify bioactive constituents released during digestion, which can provide a comprehensive assessment of the nutritional bioaccessibility of the composite gels. This study further aimed to clarify the effects of KGM and INU on the textural and nutritional properties of thermally induced composite gels and to provide a theoretical basis for the rational design of novel soft gel-based foods that combine health-promoting functionality with suitable textural properties for individuals with swallowing disorders.

## 2. Materials and Methods

### 2.1. Materials

Fresh silver carp (*H. molitrix*, *n* = 30, weight = 1.5–2.0 kg) were obtained from Dalian Shushipin market (Dalian, China). Ready-to-eat sea cucumbers (*A. japonicus*, *n* = 55, weight = 150–180 g) were purchased from Dalian Caishendao Co., Ltd. (Dalian, China). Food-grade KGM and INU were purchased from Guosheng Biological Technology Co., Ltd. (Xi’an, China). All other reagents were of analytical grade and were purchased from Shanghai Aladdin Biochemical Technology Co., Ltd. (Shanghai, China) and Shanghai Maclin Biochemical Technology Co., Ltd. (Shanghai, China).

### 2.2. Preparation of Silver Carp-Sea Cucumber (SC–SC) Composite Gel

The silver carp were slaughtered immediately, after which the heads, scales, and viscera were removed manually. The prepared fish filets were rinsed and cut into small pieces. Subsequently, the coarsely processed fish paste was ground twice with 1.5 times its weight of water using a high-speed blender (HP-SZ70, Guangdong Shunde Domeng Electric Appliance Co., Ltd., Shunde, China) for 2 min. The frozen sea cucumbers were thawed overnight at 4 °C and ground twice in an equal weight of water for 2 min each. The sea cucumber paste (moisture content, 96%) and silver carp paste (moisture content, 87%) were then mixed at a ratio of 3:7 and homogenized for 1 min using a colloid mill (LTJM-30BX, Langtong Machinery Co., Ltd., Langfang, China) [10]. The basic nutritional components in the above mixed silver carp and sea cucumber paste are as follows: moisture content: 89.7%, protein content: 11.22%, fat content: 0.86%, ash content: 0.70%, and total sugar content: 4.17%. KGM or INU was added to the prepared SC–SC blended paste at concentrations of 0%, 0.5%, 1.0%, and 1.5% (*w*/*w*). Detailed formulations of the composite pastes are provided in Table S1. The mixtures were transferred into cylindrical plastic casings (diameter = 25 mm, height = 30 mm) and heated at 90 °C for 30 min. All samples were immediately cooled in ice water for 10 min and stored overnight in a refrigerator before subsequent measurements. Before analysis, the samples were equilibrated at room temperature (RT, 25 °C) for 30 min.

### 2.3. Determination of Gel Properties

#### 2.3.1. TPA

TPA was conducted using the TMS–PRO texture analyzer (Food Technology Corporation, Sterling, VA, USA) equipped with a P/25 probe (test speed: 1.0 mm/s, compression: 50%, and trigger force: 0.1 g) at RT. The cylindrical gel samples with a height of 30 mm and a diameter of 25 mm were used for the TPA test, according to our previously reported method [11]. Six replicate samples were analyzed for each treatment group.

#### 2.3.2. Color Determination

Color measurements were performed using a chroma meter (Konica Minolta CR-410, Osaka, Japan) according to the method described by Xin et al. [12]. The whiteness (W) of each formulation was calculated and compared among treatments.

#### 2.3.3. Rheological Properties

The rheological properties of the different SC–SC composite gels were measured at RT using a rheometer (Discovery HR-1, TA Instruments, New Castle, DE, USA). Oscillatory strain sweeps were performed over a strain range of 1–100% using a 40 mm parallel plate with a 1 mm gap. Oscillatory frequency sweeps were performed over an angular frequency range of 1–300 rad/s at a constant strain of 1% within the linear viscoelastic region (LVR). The storage modulus (G′) and loss modulus (G″) were recorded to evaluate the viscoelastic properties of the composite gels. Steady-shear viscosity was measured over a shear-rate range of 0.1–100 s^−1^ [13].

#### 2.3.4. IDDSI Evaluation

The SC–SC composite gels were evaluated using the fork drip test, fork pressure test, and spoon tilt tests to determine their appropriate levels within the IDDSI framework (version 2.0, 2019). All tests were performed at RT. For the fork drip test, each sample was placed on a fork, and its flow behavior was observed. For the fork pressure test, the sample was pressed gently with the back of the fork to observe whether the sample could be easily crushed. For the spoon tilt test, sample behavior was examined as the spoon was slowly tilted. Sample flow, deformation, crush resistance, and release behavior were recorded during both tests.

#### 2.3.5. Chemical Force

The contributions of different chemical interactions were measured as previously described [14], with slight modifications. Each sample (1 g) was mixed with 10 mL of the respective extraction medium, 0.05 mol/L NaCl; 0.6 mol/L NaCl; 0.6 mol/L NaCl + 1.5 mol/L urea; 0.6 mol/L NaCl + 8 mol/L urea; 0.6 mol/L NaCl + 8 mol/L urea + 1.5 mol/L β-mercaptoethanol, as previously described, and stirred continuously at 4 °C for 1 h. The mixtures were centrifuged at 6873× *g* for 10 min at 4 °C. The contributions of the different chemical interactions were calculated based on the amount of protein solubilized in each extraction medium, as determined using the Biuret method.

#### 2.3.6. FTIR

FTIR spectra of the SC–SC composite gels were acquired as described by Zhang et al. [15] using an ATR-FTIR spectrometer (Nicolet iS50, Thermo Nicolet Corp., Madison, WI, USA). Freeze-dried samples (10 mg) were ground with a 100-fold amount of KBr to prepare pellets, which were scanned over a wavenumber range of 400–4000 cm^−1^.

### 2.4. In Vitro Digestion

The simulated in vitro digestion experiments were conducted using two separate models: an adult digestion model [16] and an elderly digestion model [17]. Simulated intestinal fluid (SIF) was used as appropriate for the respective digestion phases. The specific formulations and conditions used for the two digestion models are provided in Table S2.

The adult digestion model was conducted as follows. Samples (5.0 g) were mixed with SSF, salivary α-amylase solution, CaCl_2_, and distilled water. After the oral phase, the pH was adjusted to 3.0 using 6 mol/L HCl, and pepsin was added to achieve a final concentration of 2000 U/mL. Gastric digestion was conducted at 37 °C and pH 3.0 for 120 min. At the end of gastric digestion, the pH was adjusted to 7.0 using 1 mol/L NaOH to inactivate pepsin. Subsequently, the mixture was combined with SIF, and trypsin was added to achieve a final concentration of 100 U/mL. Intestinal digestion was conducted in a shaking water bath at 200 rpm and 37 °C for 180 min at pH 7.0. Digestion was terminated by heating the samples at 95 °C for 5 min.

Unlike the adult digestion model, the final pepsin concentration used to simulate gastric digestion in the elderly model was reduced to 1500 U/mL. Gastric digestion was conducted at 37 °C and pH 6.0 for 120 min. The final trypsin concentration was reduced to 50 U/mL, and intestinal digestion was conducted in a shaking water bath at 60 rpm for 240 min. All other procedures were performed as described for the adult digestion model.

### 2.5. Digestive Characteristics of SC–SC Composite Gel

#### 2.5.1. Digestibility

Digested samples were centrifuged at 6873× *g* for 20 min at 4 °C. The precipitates were collected and weighed [18]. Protein digestibility (PD) was calculated as follows:

$$PD\%=\left(1-\frac{m_{1}}{m_{0}}\right)\times 100\%$$

where m_0_ is the protein content of the original SC–SC composite gel (g) before digestion, and m_1_ is the protein content of the precipitate (g) after digestion.

#### 2.5.2. Amino Acid Composition

To precipitate proteins, the digesta supernatant was mixed with sulfosalicylic acid (10%, *w*/*v*) at a 4:1 (*v*/*v*) ratio and stored at 4 °C for 2 h. After centrifugation at 10,000× *g* for 15 min at 4 °C, the supernatant was filtered through a 0.22-μm membrane. Aliquots (20 μL) of the pretreated samples were injected into an amino acid analyzer (A300, MembraPure, Hennigsdorf, Germany). The concentrations of individual amino acids in the digesta were quantified by comparison with amino acid standards [19].

#### 2.5.3. Observation of Digestive Products

To compare digestion efficiency among the different groups, the digestion products were examined using atomic force microscopy (AFM; Bruker Instruments Co., Karlsruhe, Germany) as previously described [20]. Briefly, the digestion products were completely dispersed in distilled water, deposited onto freshly cleaved mica sheets, and air-dried at room temperature before molecular morphology was examined. The mica substrate was scanned in tapping mode over an area of 5.0 × 5.0 μm^2^. Surface morphology and cross-sectional profiles were analyzed using NanoScope software (NanoScope v5.30r3, Veeco, Plainview, NY, USA).

#### 2.5.4. Antioxidant Activities of Digestive Products

##### DPPH Radical Scavenging Activity (DPPH–RSA)

The 2,2-diphenyl-1-picrylhydrazyl (DPPH) radical scavenging capacity was measured as previously described [21] with minor modifications. Each digestion product (2 mL) was mixed with 2 mL of ethanolic DPPH solution (0.2 mol/L) and incubated in the dark for 30 min. Subsequently, the absorbance was measured at 517 nm. DPPH radical scavenging activity was calculated as follows:

$$DPPH\;radical\;scavenging\;capacity\;\left(\%\right)=1-\frac{A_{x}}{A_{0}}\times 100$$

where A_0_ is the absorbance of the control, and Ax is the absorbance of the samples.

##### ABTS Radical Scavenging Activity (ABTS–RSA)

The 2,2-azino-bis-(3-ethylbenzthiazoline-6-sulfonic acid) (ABTS) radical scavenging activity was determined as described by Zhang et al. [22]. A stock solution was prepared by mixing 7 mmol/L ABTS solution with 2.5 mmol/L potassium persulfate at a 1:1 (*v*/*v*) ratio. The mixture was then stored in the dark for 12 h. The stock solution was then diluted 1:50 with absolute methanol to obtain an absorbance of 0.70 ± 0.02 at 734 nm and was used as the ABTS•^+^ working solution. The sample (750 μL) was mixed with 5 mL of ABTS•^+^ working solution and incubated at RT for 45 min. ABTS radical scavenging activity was calculated as follows:

$${ABTS}^{+}\;radical\;scavenging\;capacity\;\left(\%\right)=\left(1-\frac{A_{x}}{A_{0}}\right)\times 100$$

where A_0_ is the absorbance of the control, and Ax is the absorbance of the samples.

##### Iron Reduction Ability

Ferric reducing antioxidant power (FRAP) was determined as described by Barreca et al. [23]. The acetic acid solution (0.30 mol/L, pH 3.6), 2,4,6-tripyridyl-s-triazine solution (10 mmol/L in 40 mmol/L HCl), and ferric chloride solution (20 mmol/L) were mixed at a ratio of 10:1:1 (*v*/*v*/*v*) and incubated in a water bath at 37 °C for 30 min to prepare the FRAP working solution. Subsequently, 100 μL of sample was mixed with 3 mL of the working solution and incubated at 37 °C for 8 min. Sample absorbance was measured at 593 nm. A standard curve was constructed using Trolox, and the reducing antioxidant capacity was expressed as micromoles of Trolox equivalents (TE) per gram of sample (mmol TE/g).

##### Metal Chelating Ability (MCA)

Ferrous ion chelating activity was determined as described by Buamard et al. [24]. Briefly, 2 mL of sample was mixed with 0.05 mL of FeCl_2_ solution (2 mmol/L) and incubated at RT for 10 min. Subsequently, the reaction was initiated by adding 0.2 mL of ferrozine solution (5 mmol/L). For the control, ferrozine solution was replaced with distilled water. The absorbance of both the control and test samples was measured at 562 nm. Metal ion chelating capacity was calculated as follows:

$$Metal\;ion\;chelating\;capacity\;\left(\%\right)=\left(1-\frac{A_{x}}{A_{0}}\right)\times 100$$

where A_0_ is the absorbance of the control, and Ax is the absorbance of the samples.

### 2.6. Statistical Analysis

All experiments were conducted independently in three batches, with three or six replicates. Statistical analyses were performed using SPSS Statistics version 27 (SPSS Inc., Chicago, IL, USA). Data were analyzed by one-way analysis of variance (ANOVA), followed by Duncan’s multiple range test for comparisons among means, with significance set at *p* < 0.05. Figures were prepared using Origin 2025 (OriginLab Co., Northampton, MA, USA).

## 3. Results and Discussion

### 3.1. Textural Properties of SC–SC Composite Gel

Food acceptability among individuals with dysphagia is strongly influenced by textural properties. Foods designed for this population should exhibit adequate softness and cohesiveness to facilitate bolus formation while requiring less masticatory effort, thereby reducing the risk of aspiration during swallowing [1]. As summarized in Table 1, hardness and springiness were markedly affected by KGM incorporation. The addition of KGM significantly reduced gel hardness (*p* < 0.05), with the control group exhibiting the highest value (0.333 ± 0.047 N) and the 1.0% KGM group exhibiting the lowest value (0.036 ± 0.004 N). In contrast, INU supplementation had no significant effect on hardness (*p* > 0.05). The pronounced softening effect of KGM made springiness undetectable in the 0.5%, 1.0%, and 1.5% KGM groups. These gels were too soft to recover their original shape after compression, and the corresponding springiness values are therefore marked as not detectable in Table 1. Excessive hardness in composite gels can prolong oral processing and swallowing; the KGM-induced reduction in hardness may be attributed to the incorporation of non-starch polysaccharide chains into the protein gel matrix, which weakens protein–protein interactions and promotes the formation of a looser polymeric network [25]. Consistent with our previous findings, low concentrations of INU had little effect on surimi gel firmness, which may be associated with its degree of polymerization and concentration [26]. Cohesiveness, an indicator of the internal bonding strength of the gel network, increased with the addition of KGM and 1.0% INU, by approximately 46.52% and 4.06%, respectively, relative to the control group (0.493 ± 0.087). Adequate cohesiveness maintains the integrity of the bolus during oral processing, which may reduce food fragmentation during swallowing and subsequent entry into the airway, thereby improving bolus safety and reducing the risk of choking [27,28]. Notably, chewiness did not vary in parallel with hardness: although KGM markedly decreased gel hardness, the KGM-treated groups exhibited significantly higher chewiness than the control (*p* < 0.05), which was predominantly driven by their markedly increased cohesiveness rather than overall gel strength (Table 1). Gels with lower chewiness are generally easier for individuals with swallowing impairments to consume. Adhesiveness, defined as the resistance to separation between the gel surface and the oral mucosa, decreased as the KGM or INU concentration increased, consistent with the apparent viscosity trends shown in Figure 1B. INU was particularly effective in reducing adhesiveness; at 0.5% INU, adhesiveness decreased to 0.183 ± 0.054, compared with 0.210 ± 0.029 in the control group, although only the 1.5% KGM treatment (0.177 ± 0.050) produced a significant reduction. The generally low adhesiveness of the KGM- and INU-supplemented gels may facilitate the formation of a cohesive bolus during oral processing, which is beneficial for safe swallowing in individuals with dysphagia. Overall, these findings indicate that KGM and INU can effectively modulate gel network structure and modify textural characteristics. Nevertheless, the significantly increased chewiness of the KGM-treated gels indicates that excessive KGM supplementation may increase masticatory difficulty; accordingly, the suitability of these composite gels for dysphagia management should be evaluated comprehensively in combination with IDDSI classification and other swallowing-related parameters.

### 3.2. Color Characteristics of SC-SC Composite Gel

Color is an important sensory attribute that influences consumer acceptance, particularly among individuals with dysphagia, for whom poor visual appeal may reduce appetite and dietary intake [29]. As shown in Figure 1A, KGM incorporation significantly decreased the *a^∗^*, *b^∗^*, and *L^∗^* values of the composite gels (*p* < 0.05). Among these parameters, *L^∗^* showed the greatest change, with the 0.5% KGM group exhibiting the lowest value, representing a 27.79% reduction relative to the control group. Concurrently, the *b^∗^* value shifted from positive to negative, indicating a change toward a bluer color. This effect may be attributed to interactions between the hydroxyl groups of KGM and the protein matrix, which alter the gel network’s pore structure and reduce light reflection and scattering, thereby decreasing overall lightness and color saturation [29]. In contrast, INU supplementation also significantly decreased the *a^∗^* and *L^∗^* values (*p* < 0.05), although the changes were less pronounced than those observed with KGM. The greatest reduction in *L^∗^* among the INU-treated groups occurred at 1.5% INU, corresponding to a 9.63% decrease relative to the control. INU addition also significantly increased the whiteness index of the composite gels (*p* < 0.05), regardless of concentration, with an average increase of 5.32% relative to the control group. This whitening effect may result from the dissolution and distribution of INU within the interstitial spaces of the gel network, potentially enhancing interfacial light reflection and increasing whiteness [30]. Overall, supplementation with KGM or INU modified the color characteristics of the composite gels and contributed dietary fiber, which may improve the gels’ visual and nutritional suitability for the target population.

### 3.3. Rheological Analysis of SC-SC Composite Gel

The effects of different KGM and INU concentrations on the apparent viscosity of the composite gels as a function of shear rate are shown in Figure 1B. All seven formulations exhibited pseudoplastic, shear-thinning behavior, characterized by a reduction in apparent viscosity as shear rate increases. This rheological property is advantageous for foods intended for individuals with dysphagia because it allows viscosity to decrease during oropharyngeal and esophageal flow, potentially facilitating safer swallowing [31]. It should be emphasized that the shear rate range selected for rheological testing needs to match the physiological shear rate of human swallowing; the use of excessively high shear rates in rheological evaluations has been considered less physiologically relevant when designing foods for frail elderly individuals or individuals with dysphagia [32]. Accordingly, apparent viscosity was compared among formulations at a fixed shear rate of 1 s^−1^. KGM markedly increased viscosity from 81.6586 Pa·s in the control to 184.514 Pa·s, 139.304 Pa·s, and 138.332 Pa·s in the 0.5%, 1.0%, and 1.5% KGM groups, respectively. This thickening effect may be attributed to the formation of a viscoelastic matrix through interactions between KGM molecules and gel proteins, which increases resistance to flow and rearranges the spatial structure of the gel network [28]. These findings are consistent with the texture profile analysis results presented in Table 1. In contrast, INU supplementation reduced viscosity to 55.1498 Pa·s, 63.4943 Pa·s, and 39.0717 Pa·s in the 0.5%, 1.0%, and 1.5% INU groups, respectively. This decrease in viscosity suggests weakening of the protein network, since the incorporation of INU interferes with protein cross-linking and produces a looser gel structure with lower resistance to flow [26].

To further characterize the viscoelastic properties and gel network, the storage modulus (G′) and loss modulus (G″) were evaluated (Figure 1C,D). Across the measured linear viscoelastic region, G′ remained higher than G″ for all composite gels, indicating predominantly elastic character and confirming the solid-like gel properties of the systems. These results indicate that the incorporation of either KGM or INU maintained the overall gel structure of the composites. The INU-containing samples exhibited considerably higher G′ values than the corresponding KGM-containing samples at identical addition levels. At low KGM or INU concentrations, both G′ and G″ showed a decreasing trend with increasing angular frequency, suggesting progressive weakening or disruption of the gel network associated with reduced intermolecular physical interactions [33]. In contrast, at higher KGM or INU concentrations, G′ and G″ increased with frequency, indicating greater resistance to deformation and a more stable internal network structure [34]. Compared with liquid diets, soft elastic gels with these rheological characteristics may be more suitable for individuals with dysphagia because they can provide greater bolus cohesiveness and potentially reduce the risks of aspiration and coughing during swallowing. Overall, modulating the textural and rheological properties through the incorporation of KGM or INU appears to be a promising approach for developing SC–SC composite gels suitable for dysphagia management.

### 3.4. IDDSI Analysis of SC–SC Composite Gel

In the fork drip test, the flow behavior of each sample through the spaces between the fork tines was visually assessed, as shown in Figure 2. Samples without KGM remained firmly on the fork without observable dripping, indicating a consistency above IDDSI level 4 and requiring further assessment to distinguish among levels 5–7, which are suitable for individuals with dysphagia who retain some masticatory ability. In contrast, KGM-containing formulations slowly passed through the fork tines and formed a short tail beneath the fork, consistent with the criteria for level 4 (pureed/extremely thick) foods, which are intended for individuals with substantially impaired swallowing function.

The spoon tilt test was used to evaluate the adhesiveness and cohesiveness of the gel systems [35]. When the spoon was tilted, samples containing 0.5%, 1.0%, and 1.5% KGM largely retained their integrity on the spoon surface and exhibited limited detachment, indicating relatively strong cohesive and adhesive properties. This observation was consistent with the increased cohesiveness of the KGM-containing gels observed in the texture profile analysis. In contrast, the control and INU-supplemented samples (0.5%, 1.0%, and 1.5%) maintained their molded shapes on the inclined spoon while exhibiting comparatively lower adhesion to the spoon surface.

In the fork pressure test, all KGM-containing gels were readily compressed and deformed under minimal pressure, leaving no obvious lumps or particulate residues and displaying clear fork-imprint patterns on their surfaces. These characteristics were consistent with the IDDSI level 4 (pureed/extremely thick) classification and support their potential suitability for individuals with severely limited chewing ability. In contrast, the control and INU-containing gels required considerably greater pressure to break apart and did not recover their original shapes after compression. These characteristics were consistent with level 6 (soft and bite-sized) foods, which are more appropriate for individuals with dysphagia who retain some masticatory function.

### 3.5. Chemical Interactions Analysis of SC-SC Composite Gel

As shown in Figure 3A, hydrogen bonding and hydrophobic interactions were the predominant forces contributing to stabilization of the gel network, while KGM and INU exerted distinct effects on intermolecular interactions within the composite gels. As KGM concentration increased, the ionic bond content initially decreased and then increased relative to the control, reaching its highest value (0.019 ± 0.0089 mg/mL) at 1.5% KGM. At a low KGM concentration (0.5%), the hydroxyl groups of the polysaccharide may compete with polar protein groups for cation-binding sites, thereby disrupting electrostatic crosslinks between protein molecules. At KGM concentrations above 1.0%, increased polysaccharide levels may promote protein aggregation and re-exposure of charged residues, thereby facilitating the reformation of ionic interactions. Hydrogen bond content increased significantly (*p* < 0.05) following KGM addition, consistent with the FTIR results shown in Figure 3B. As a neutral, water-soluble dietary fiber, KGM contains abundant hydroxyl groups that can form hydrogen bonds with the carboxyl and amino groups of protein subunits, potentially delaying protein denaturation and promoting exposure of reactive functional groups [36]. Hydrophobic interactions exhibited a biphasic pattern, initially decreasing and subsequently recovering, with the value in the 1.5% KGM group (0.053 ± 0.0073 mg/mL) approaching that of the control group (0.057 ± 0.0027 mg/mL). This pattern may reflect masking of hydrophobic protein regions at low KGM concentrations, whereas higher concentrations may promote protein aggregation and the re-exposure of previously buried hydrophobic groups. Disulfide bond content increased significantly (*p* < 0.05), which may be attributed to KGM-induced protein unfolding that facilitates sulfhydryl oxidation and increases covalent crosslinking. No significant difference was observed between the 1.0% (0.028 ± 0.0037 mg/mL) and 1.5% (0.026 ± 0.0037 mg/mL) KGM groups, suggesting that exposure of free thiol groups within the protein structure approached saturation at 1.0% KGM. Above this concentration, fewer oxidizable sulfhydryl groups may remain available for additional disulfide bond formation, while the greater viscosity associated with higher polysaccharide concentrations may restrict molecular contact and limit further covalent crosslinking.

In contrast, INU supplementation did not significantly alter ionic bond content (*p* > 0.05), indicating relatively weak electrostatic interactions between INU and protein molecules. Hydrogen bond content tended to increase, although the difference was not significant. The hydroxyl groups of INU can interact with carboxyl and amino groups of proteins through hydrogen bonding, although its molecular characteristics may limit the extent of these interactions. Competition with water molecules for hydrogen-bonding sites in the aqueous environment may further restrict formation of the hydrogen-bond network, resulting in only modest changes [37]. Hydrophobic interactions were significantly lower than those of the control (*p* < 0.05) following INU addition and exhibited a concentration-dependent pattern, with the highest value among the INU treatments observed at 1.0% INU (0.034 ± 0.0022 mg/mL). At INU concentrations of 0.5–1.0%, moderate protein unfolding may expose previously buried hydrophobic residues and partially strengthen hydrophobic associations within the INU-treated systems. Disulfide bond content also increased significantly (*p* < 0.05), reaching its highest value at 0.5% INU (0.0017 ± 0.0012 mg/mL), suggesting that INU may promote spatial rearrangement of protein molecules and increase contact between sulfhydryl groups, thereby favoring disulfide bond formation. Further increases in INU concentration, however, were associated with a decline in disulfide bond content. Adsorption of excess polysaccharide molecules onto protein surfaces may generate steric hindrance, limiting intermolecular contact between free thiol groups and suppressing oxidative crosslinking [38].

### 3.6. FTIR Analysis of SC–SC Composite Gel

FTIR spectroscopy was employed to elucidate the intermolecular interactions within the SC–SC composite gels containing different concentrations of KGM and INU, as shown in Figure 3B. The spectrum of the control group exhibited two characteristic absorption bands at 1576.52 cm^−1^ and 960.38 cm^−1^, which were assigned to the stretching vibration of C–N bonds within the protein backbone and the coupled vibrations of C–O and C–C groups in the polysaccharide chains, respectively [39,40]. A broad and intense absorption band centered at approximately 3400 cm^−1^ was observed in all samples and was attributed to O–H stretching vibrations, reflecting the hydrogen-bonding network involving bound water and hydroxyl groups within the system [41].

Following the incorporation of KGM or INU, notable changes in the wavenumber of characteristic peaks at approximately 3400 cm^−1^ and 1576 cm^−1^ were observed, suggesting complex physicochemical interactions between the weak gel matrix and the added polysaccharides during synergistic gelation [42]. Specifically, as the KGM/INU concentration progressively increased from 0% to 1.5%, the peak initially located at 3427.85 cm^−1^ shifted to 3413.87, 3395.07, 3423.99, 3401.82, 3414.35, and 3414.87 cm^−1^, respectively. Concurrently, the band at 1576.52 cm^−1^ shifted to 1582.31, 1585.20, 1582.31, 1589.06, 1586.65, and 1585.20 cm^−1^. These frequency shifts provide strong evidence that the formation of the composite gel network is governed by a combination of hydrogen bonding and hydrophobic interactions [43,44]. In addition, broadening of the coupled C–O/C–C vibration band at 960.38 cm^−1^ further supports the presence of hydrogen-bonding interactions between KGM/INU and the protein–polysaccharide weak gel matrix [45]. These spectral variations indicate that the molecular assembly of the composite gels is mediated by multiple noncovalent forces, which ultimately influence the structural and functional properties of the resulting products.

### 3.7. Digestibility of SC–SC Composite Gel

Dysphagic patients frequently experience concomitant declines in digestive function, often accompanied by other diet-related comorbidities, thereby requiring foods that are both texturally appropriate and readily digestible. In the adult simulated gastrointestinal system (Figure 4A), the three KGM-supplemented groups, together with the 0.5% INU and 1.5% INU groups, exhibited significantly higher gastric digestibility than the remaining formulations (*p* < 0.05). In contrast, the control (16.23 ± 3.61%) and 1.0% INU (17.05 ± 3.81%) groups showed relatively low gastric digestibility. The incorporation of INU may have altered the intermolecular interactions and microstructural organization of the protein–polysaccharide network, thereby affecting the accessibility of the gel matrix to digestive fluids and enzymes. At 0.5% INU, the relatively low polysaccharide content may have caused only limited structural modification, whereas at 1.5% INU, the higher content may have reorganized the network and improved the access of digestive fluids and enzymes. These structural differences may partly explain the variation in gastric digestibility among the INU-containing gels. This reduced susceptibility to gastric digestion may be attributed to the densely crosslinked gel architecture, which restricts the contact area between digestive fluids and the gel matrix and limits interfacial enzyme adsorption [45]. Regarding intestinal digestibility, the 1.5% INU group showed significantly higher values than the other groups (*p* < 0.05). Notably, all composite gels achieved intestinal digestibility exceeding 90% in the adult model, indicating highly efficient nutrient release and hydrolysis under simulated adult physiological conditions.

In the geriatric in vitro system, the 0.5% KGM and 1.0% KGM groups exhibited significantly higher gastric digestibility than the other samples (*p* < 0.05). Consistent with findings from the adult model, the control (15.06 ± 2.99%) and 1.0% INU (18.07 ± 3.81%) groups again exhibited the lowest gastric digestibility, reinforcing the observation that compact gel networks can impede proteolytic breakdown across diverse digestive environments. This consistent trend suggests that the structural organization of the composite gel matrix may contribute to differences in its susceptibility to gastric digestion under both simulated adult and elderly conditions. Interestingly, the control group exhibited higher intestinal digestibility than all KGM/INU-supplemented gels, suggesting that the incorporation of these hydrocolloids moderately reduced the extent of intestinal hydrolysis. This effect may be attributed to the formation of cross-linked structures arising from changes in the protein–polysaccharide network, which could reduce the accessibility of protein substrates to digestive enzymes. Similar interactions between proteins and polysaccharides may occur, which can obscure enzyme-cleavable sites and thereby reduce proteolytic enzymes’ access to their substrates under both adult and elderly simulated gastrointestinal conditions [18]. The overall digestibility of all composite gels exceeded 90% in both the adult and elderly simulated digestion models, demonstrating their favorable digestive compatibility. Taken together, the developed KGM/INU composite gels exhibited high overall digestibility while retaining appropriate gel properties, suggesting their potential as bioaccessible food matrices for individuals with dysphagia.

### 3.8. FTIR Analysis of SC–SC Composite Gel Digestion Products

In the infrared spectra shown in Figure 4B,C for adults and elderly digestion products, the absorption peaks at 2800–3100 cm^−1^ represent the symmetric/asymmetric vibrations of C-H bonds (such as –CH_3_, =CH_2_), reflecting the structural characteristics of the aliphatic side chains of amino acids. After gastric digestion, the peak shape and intensity in this region showed no significant change, indicating that the aliphatic structure remained relatively stable during the digestion. A new C-N absorption peak appeared at 1350–1450 cm^−1^ after gastrointestinal digestion, indicating that the active site of pepsin acts on the C-N bond. Meanwhile, the absorption peak of C-C at 1066 cm^−1^ weakened, suggesting that C-C bonds are a site of action for trypsin and that some peptide chains may be further broken down into amino acids [43]. Although the main characteristic absorption peaks remained stable after digestion, some differences in absorption wavelengths and intensities were still observed among the digestion products of adults and the elderly. After hydrolysis by pepsin and trypsin, the tertiary structure of proteins was disrupted, the secondary structure became disordered, peptide bonds and some C-H and C-C bonds were broken, and the proteins were ultimately hydrolyzed into amino acids [46]. This process is consistent with the digestibility results (Section 3.7), confirming that the composite gel can be effectively decomposed during digestion, thereby providing absorbable nutrients for people with swallowing difficulties.

### 3.9. Amino Acid Composition of SC–SC Composite Gel Digestion Products

The amino acids in the adult and elderly digests of the SC–SC composite gels are shown in Figure 4D. In the adult digestion model, the control group exhibited a comparatively low total amino acid (TAA) content of 47.30 mg/100 g. Among the essential amino acids in the control group, lysine was the most abundant (4.39 mg/100 g), serving as an important substrate for skeletal muscle protein synthesis and contributing to tissue repair and the maintenance of immune homeostasis. Methionine was present at the lowest concentration (1.27 mg/100 g); this sulfur-containing amino acid participates in methylation reactions and biological detoxification, contributes to the regulation of sulfur-containing compound metabolism, and may protect against oxidative damage in elderly individuals [2]. Among the nonessential amino acids, glutamic acid was present at the highest concentration (7.26 mg/100 g) and contributed to muscle protein accretion, wound healing, and immune function, making it a functionally relevant amino acid in nutritional formulations for elderly individuals [17].

Following KGM and INU supplementation, the amino acid profiles of the adult digests changed to varying degrees. The TAA contents followed the descending order 1.0% KGM > 0.5% KGM > 1.5% KGM >1.5% INU > 0.5% INU > 1.0% INU. This trend reflects the distinct regulatory effects of KGM and INU on protein hydrolysis during in vitro digestion. This pattern suggests that KGM addition may protect protein substrates during digestion, thereby promoting the amino acid release. In the elderly digestion model, the 1.5% KGM group exhibited the highest TAA content (56.70 mg/100 g). Compared with the adult digestion results, overall amino acid release after simulated elderly digestion was consistently higher for the same gel formulations. This difference originates from the distinct gastrointestinal environmental parameters between adult and elderly digestion systems, such as pH, which alters the charge state of protein molecules and further modulates the polysaccharide–protein interactions. This phenomenon is regulated by the pH-dependent interaction between polysaccharides and proteins. The simulated gastric environment for the elderly has a relatively higher pH, which alters the hydrogen-bonding and noncovalent interactions between polysaccharides and proteins, facilitates the dissociation of protein–polysaccharide complexes, and alleviates the spatial hindrance effect on hydrolysis mediated by gastric protease [46]. It is worth noting that in both digestion models, the TAA content of the digestion products in the 1.0% INU sample group decreased. When the INU concentration increased to 1.0%, the increased number of INU molecules might promote hydrogen-bonding and other noncovalent interactions between INU and protein side chains, gradually forming protein–polysaccharide complexes. This concentration-dependent complexation may create a spatial barrier on the protein surface, potentially hindering pepsin binding to the substrate and reducing the degree of hydrolysis [38]. In summary, in the context of food development for the elderly, excessive INU addition may promote the formation of protein–polysaccharide complexes that reduce protein bioavailability, whereas appropriate KGM supplementation may achieve a balance between desirable gel texture and efficient amino acid release.

### 3.10. Microstructure Observation of SC–SC Composite Gel Digestion Products

AFM was used to visualize the microstructural characteristics of digestion products obtained from the composite gels under both adult and elderly simulated digestion conditions, with representative 2D and 3D topographic images shown in Figure 5A and Figure 5B, respectively. In the adult digestion model, micrographs of the control group revealed several irregularly shaped and heterogeneously distributed protein aggregates in both the 2D and 3D images. This morphological heterogeneity may be attributed to thermal expansion or cleavage of myofibrillar protein side chains during digestion, which subsequently promotes self-association into larger, nonuniform protein particles [20]. Incorporation of low-to-moderate KGM concentrations (0.5–1.0%) and a high INU concentration (1.5%) resulted in more regular and homogeneous aggregates, suggesting that these concentrations favor the formation of more continuous and uniform digestion residues. The Rq value of the 1.5% KGM group (2.73 ± 0.85 nm) was slightly higher than that of the control group (2.11 ± 0.54 nm), indicating a modest increase in surface irregularity. Moreover, the 1.5% INU group exhibited a lower Rq value (2.03 ± 1.14 nm), whereas the 1.0% INU group showed a substantially higher Rq value (4.49 ± 0.05 nm), indicating a marked increase in surface roughness.

The elderly system morphological characteristics were generally similar to those observed in the adult system, suggesting that differences in digestion conditions did not fundamentally alter the microstructural features of the control digests. Supplementation with medium-to-high KGM concentrations (1.0–1.5%) and low-to-moderate INU concentrations (0.5–1.0%) produced more ordered and uniform digestion products. The Rq values of the 1.0% and 1.5% KGM groups decreased to 0.30 ± 0.13 nm and 0.74 ± 0.28 nm, respectively, both of which were markedly lower than the control value (2.76 ± 0.71 nm). Similarly, the 0.5% and 1.0% INU groups showed lower Rq values of 2.63 ± 1.18 nm and 1.49 ± 0.54 nm, respectively, whereas the 1.5% INU group exhibited a pronounced increase to 4.12 ± 0.06 nm.

As a quantitative measure of root-mean-square surface roughness, Rq provides useful information regarding topographic changes induced by enzymatic hydrolysis. The observed increase in surface roughness during digestion may be attributed to the preferential degradation of randomly oriented amorphous edge regions by proteolytic enzymes [46]. Cross-sectional profile analysis of the control digests revealed a loosely packed structure with prominent protrusions and depressions, which may be readily accessible to enzymatic attack. In contrast, following digestion in adults and elderly individuals, the KGM/INU-supplemented samples exhibited sharp, ridge-like features indicative of a more compact, denser architecture, potentially reducing accessibility to digestive enzymes, consistent with the digestibility results discussed in Section 3.7. These findings demonstrate that the addition of KGM or INU effectively modulates the micromorphology and surface roughness of the digestion products. Low-to-moderate concentrations of either polysaccharide tend to promote the formation of more homogeneous, readily digestible residues, whereas excessive concentrations may induce structural densification, thereby reducing overall digestive efficiency.

### 3.11. Antioxidant Activities of SC–SC Composite Gel Digestion Products

#### 3.11.1. DPPH–RSA

The DPPH radical scavenging rate is a well-established indicator for evaluating the antioxidant capacity of food matrices, reflecting the abundance and bioactivity of antioxidant peptides and other active constituents in digestion products [18]. The antioxidant activity measured in this study mainly originates from protein-derived peptides generated during gastrointestinal hydrolysis, while KGM and INU may indirectly modulate peptide release through altering gel network structure. As illustrated in Figure 6A, during the gastric digestion phase of the adult model, the 1.0% KGM (72.54 ± 0.22%) and 1.5% KGM (74.40 ± 0.85%) groups exhibited significantly higher DPPH radical scavenging rates than the other formulations (*p* < 0.05), demonstrating that moderate-to-high KGM concentrations effectively enhance the antioxidant potential of gastric digests. Furthermore, under elderly gastric digestion conditions, the 0.5% INU group exhibited the highest scavenging rate (62.80 ± 0.77%), significantly higher than those of all other treatments (*p* < 0.05), suggesting that a low INU concentration is more favorable for promoting antioxidant activity under such conditions.

Following simulated intestinal hydrolysis in the adult model, the 1.5% INU group exhibited the highest DPPH radical scavenging rate (67.44 ± 1.23%), significantly exceeding those of the remaining groups (*p* < 0.05). This result suggests that a high INU concentration promotes the generation or release of antioxidant components during intestinal digestion. In the elderly intestinal model, the 0.5% INU group exhibited the highest DPPH radical-scavenging rate (55.84 ± 1.22%), consistent with the trend observed during the gastric phase of the same model. The markedly enhanced DPPH radical scavenging capacity observed after gastric digestion in both the adult and elderly models may be attributed to the presence of abundant hydrophobic residues, such as proline, alanine, and leucine, in the digested peptide fractions. The molecular weight distribution of released peptides also contributes to radical scavenging capacity; shorter peptide fragments often exhibit stronger DPPH scavenging ability. These hydrophobic residues are known to exhibit greater affinity for the lipophilic DPPH radical, thereby enhancing overall scavenging efficiency [18]. In contrast, some intestinal digests exhibited lower antioxidant activity than their gastric counterparts. This reduction may result from the hydrolytic action of trypsin, which can cleave antioxidant peptide sequences, thereby partially diminishing their radical scavenging capacity [47]. Peptide amino acid sequence is another key determinant, as cleavage may disrupt active sequence motifs responsible for radical quenching. These findings demonstrate that incorporating KGM and INU effectively modulates the antioxidant properties of the digestion products. Moderate-to-high KGM concentrations confer greater antioxidant activity in adult gastric digests, whereas low INU concentrations appear to be more advantageous under elderly digestive conditions.

#### 3.11.2. ABTS–RSA

ABTS–RSA is used to evaluate the ability of substances to donate hydrogen atoms and interrupt oxidative chain reactions, thereby reflecting the overall antioxidant potential of bioactive compounds present in digestion products [48]. Similar to the DPPH assay, the antioxidant response primarily comes from bioactive peptides released from the protein matrix. As shown in Figure 6B, during the gastric phase of adult digestion, the 0.5% INU group exhibited a significantly higher ABTS•^+^ radical scavenging capacity (81.84 ± 0.30%) than the other formulations (*p* < 0.05), representing a 5.37% increase relative to the control group. In the elderly model, both the control group (85.99 ± 0.25%) and the 0.5% INU group (86.81 ± 0.30%) exhibited significantly greater ABTS•^+^ radical scavenging capacities than the other treatments (*p* < 0.05). Following simulated intestinal hydrolysis, the ABTS•^+^ radical scavenging activities of all digestion products increased during the intestinal phase. In the adult digestion model, the KGM-supplemented groups (0.5–1.5%) showed significantly higher scavenging rates than the other groups (*p* < 0.05), with values of 92.24 ± 0.08%, 92.41 ± 0.08%, and 92.41 ± 0.08%, respectively. Moreover, in the elderly intestinal digestion model, the 1.5% KGM group exhibited the highest ABTS•^+^ radical scavenging rate (92.53 ± 0.08%). These results demonstrate that KGM significantly enhances the antioxidant capacity of intestinal digests.

The consistently strong ABTS•^+^ radical scavenging activity observed in both adult and elderly gastric and intestinal digests may be attributed to the release of oligopeptides, aromatic amino acids, and free amino acids resulting from proteolytic hydrolysis of the composite gels, all of which are known to contribute to enhanced antioxidant activity [6]. Aromatic amino acid residues (tyrosine, tryptophan, phenylalanine) within peptide chains are critical for donating hydrogen to neutralize ABTS radicals. Furthermore, the incorporation of KGM or INU appears to modulate protease cleavage sites, thereby facilitating the release of a greater abundance of antioxidant peptides and enhancing the overall radical scavenging capacity.

#### 3.11.3. FRAP

The FRAP assay measures the ability of antioxidants to reduce Fe^3+^ to Fe^2+^ and serves as a reliable indicator of the electron-donating ability and overall reducing potential of bioactive compounds in digestion products (Figure 6C). During the gastric digestion phase of the adult model, the 0.5% INU group exhibited a significantly higher FRAP value (3.80 ± 0.07 mmol TE/g) than the other treatments (*p* < 0.05), indicating that a low INU concentration effectively enhances the reducing capacity of gastric digests. Meanwhile, in the elderly model, both the 0.5% INU (4.29 ± 0.40 mmol TE/g) and 1.5% INU (4.06 ± 0.05 mmol TE/g) groups exhibited FRAP values significantly higher than those of the remaining treatments (*p* < 0.05), suggesting that INU supplementation markedly improves reducing power under elderly gastric digestion conditions. All digestion products exhibited higher FRAP values during the intestinal phase than during the corresponding gastric phase. In the adult model, the 1.5% KGM group exhibited the highest FRAP value (8.04 ± 0.29 mmol TE/g), representing a 1.32-fold increase relative to the control group. The differences in FRAP values following adult and elderly in vitro digestion may be primarily attributed to the unfolding of protein structures during proteolysis, which exposes amino acid residues that can interact with ferric ions and facilitate their reduction to the ferrous form [48]. Moreover, the greater degree of hydrolysis following intestinal digestion may reduce steric hindrance around antioxidant groups, allowing them to exert their electron-donating functions more effectively and thus increasing the overall FRAP values [47]. The magnitude of reducing potential is closely linked to the extent of protein hydrolysis, showing consistent trends with the digestibility results in Section 3.7. This enhanced reducing potential is consistent with the digestibility profiles discussed in the preceding sections, further confirming that the incorporation of KGM and INU modulates textural and rheological properties and positively influences the functional antioxidant capacity of the composite gel systems.

#### 3.11.4. MCA

MCA, typically assessed by the Fe^2+^ chelation rate, is an important parameter for evaluating antioxidant potential because it can suppress free radical generation by sequestering pro-oxidative metal ions, thereby interrupting metal-catalyzed oxidative reactions [49]. As shown in Figure 6D, the 1.0% INU (36.46 ± 0.82%) and 1.5% INU (37.52 ± 0.59%) groups exhibited significantly higher Fe^2+^ chelating capacities than the other formulations during adult gastric digestion (*p* < 0.05). These results indicate that higher INU concentrations effectively enhance the metal-binding activity of gastric digests. However, in the elderly gastric digestion model, the control group exhibited the highest Fe^2+^ chelation rate (53.63 ± 0.41%), which was significantly higher than those of all other treatments (*p* < 0.05). This finding suggests that, under elderly gastric conditions, protein hydrolysates derived from the unfortified gel may more readily expose chelating residues, thereby conferring greater metal-binding capacity.

In the elderly intestinal digestion model, the 1.0% KGM (58.48 ± 0.16%) and 1.5% KGM (57.95 ± 0.09%) groups exhibited significantly higher Fe^2+^ chelating capacities than the other treatments (*p* < 0.05), representing increases of 2.54-fold and 2.52-fold, respectively, relative to the control group. The effect of protein hydrolysis on chelating properties is governed by two complementary mechanisms. On the one hand, enzymatic cleavage exposes chelating residues, such as aspartic acid, glutamine, and histidine, thereby enhancing metal-binding affinity. On the other hand, excessive proteolysis may cleave or alter these critical residues, thereby reducing chelating activity. The content of basic amino acids including arginine and lysine also improves metal ion chelating ability, which is supported by our amino acid composition data. Previous studies have also reported that greater chelating activity of hydrolysates is positively associated with high arginine and lysine contents [22], consistent with the amino acid composition data obtained in the present study. Furthermore, the iron-chelating activity of digestion products is influenced by multiple factors, including protein source, enzyme specificity, and hydrolysis conditions, highlighting the complexity of predicting antioxidant functionality solely from digestibility parameters [22].

## 4. Conclusions

This study demonstrates that incorporating KGM and INU effectively modulates intermolecular forces in composite protein gels, with hydrogen bonding and hydrophobic interactions predominantly enhanced. While maintaining an elastic-dominated gel network, the addition of these hydrocolloids significantly reduced gel hardness and improved cohesiveness, thereby facilitating the formation of safely swallowable boluses better suited to the oral processing requirements of individuals with dysphagia. Notably, KGM supplementation darkened the gel color and increased food residue retention on spoons, which may increase the risk of aspiration. In contrast, INU addition produced a brighter appearance in the composite gels and reduced spoon retention, potentially lowering the likelihood of aspiration. According to the IDDSI framework, KGM-containing modified composite gels were classified as level 4 (pureed/extremely thick) foods, whereas INU-formulated gels without KGM were classified as level 6 (soft and bite-sized) foods, with both groups demonstrating favorable suitability for swallowing. Furthermore, systematic evaluation of digestibility under simulated adult and elderly gastrointestinal conditions showed that all composite gels underwent extensive hydrolysis, accompanied by reduced protein aggregation, looser microstructural features in the digesta, and enhanced antioxidant capacities. These findings indicate that the digestion process is efficient and responsive to the distinct physiological characteristics represented by the two age-specific digestion models. The digestion products contained seven essential amino acids, with particularly high levels of lysine and glutamic acid. Moreover, the digests exhibited significantly enhanced DPPH-RSA, ABTS-RSA, FRAP, and MCA, which may be attributed to the generation and release of oligopeptides, aromatic amino acids, and hydrophobic residues during proteolysis. This study provides a comprehensive characterization and rationale for the design of foods intended for individuals with dysphagia by elucidating the gelation properties, swallowing suitability, and digestive behavior of KGM/INU-modified composite gels. These findings provide a foundation for the future development of texture-modified, nutritionally fortified dietary products tailored to the needs of individuals with swallowing disorders. It should be noted that this study was limited to simulated gastrointestinal digestion and did not assess nutrient absorption or swallowing performance in older adults with dysphagia, which represents an important limitation of the present study. Further in vivo and human studies are needed to validate these aspects.

## Figures and Tables

**Figure 1:**
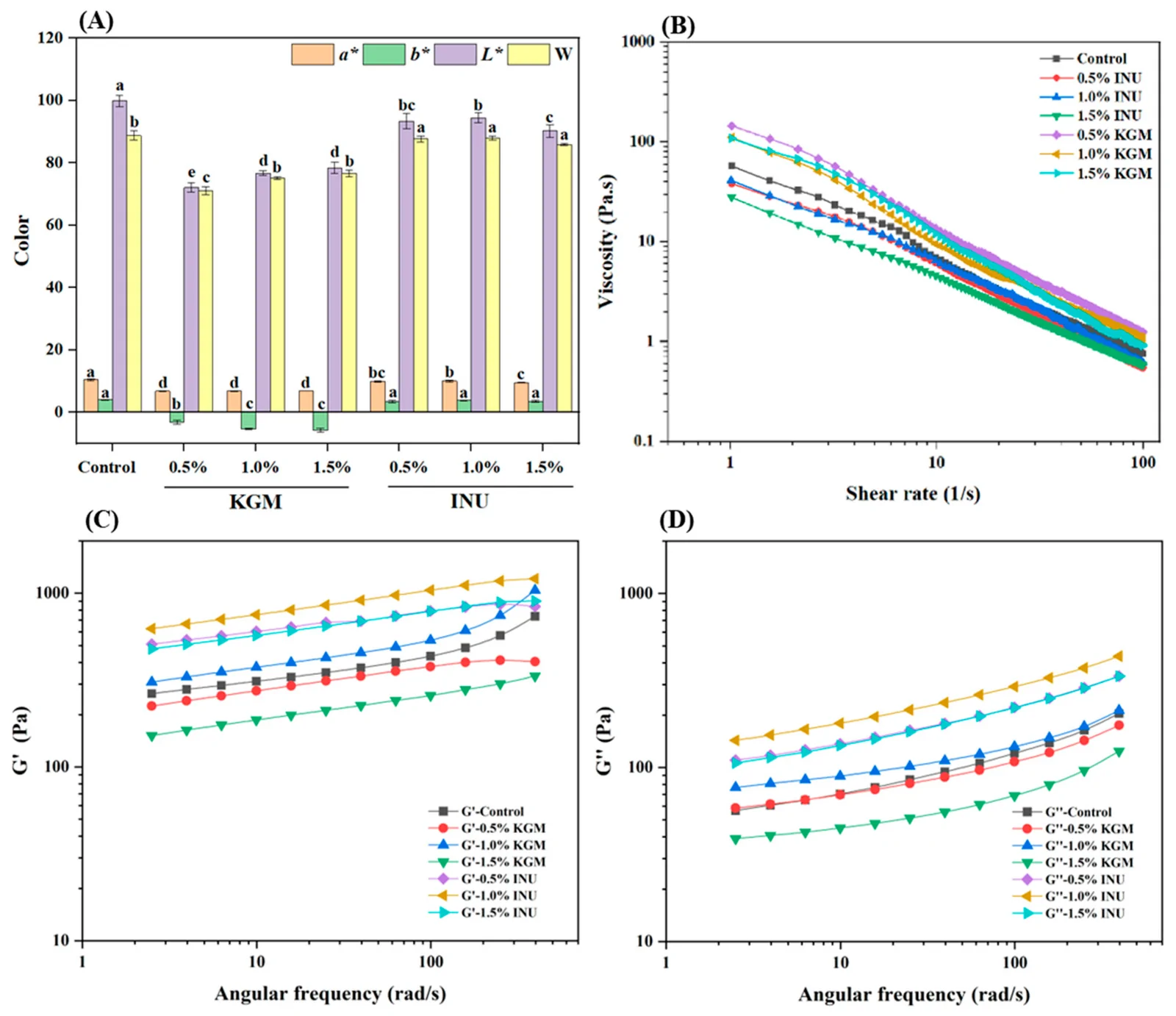
The color characteristics (**A**), rheological behaviors (viscosity (**B**), G′ (**C**) and G″ (**D**)) of SC-SC composite gels with different additions of KGM and INU. Note: Different lowercase letters (a–e) within the column indicate significant differences between groups (*p* < 0.05). G′: storage modulus; G″: loss modulus. KGM: konjac glucomannan; INU: inulin.

**Figure 2:**
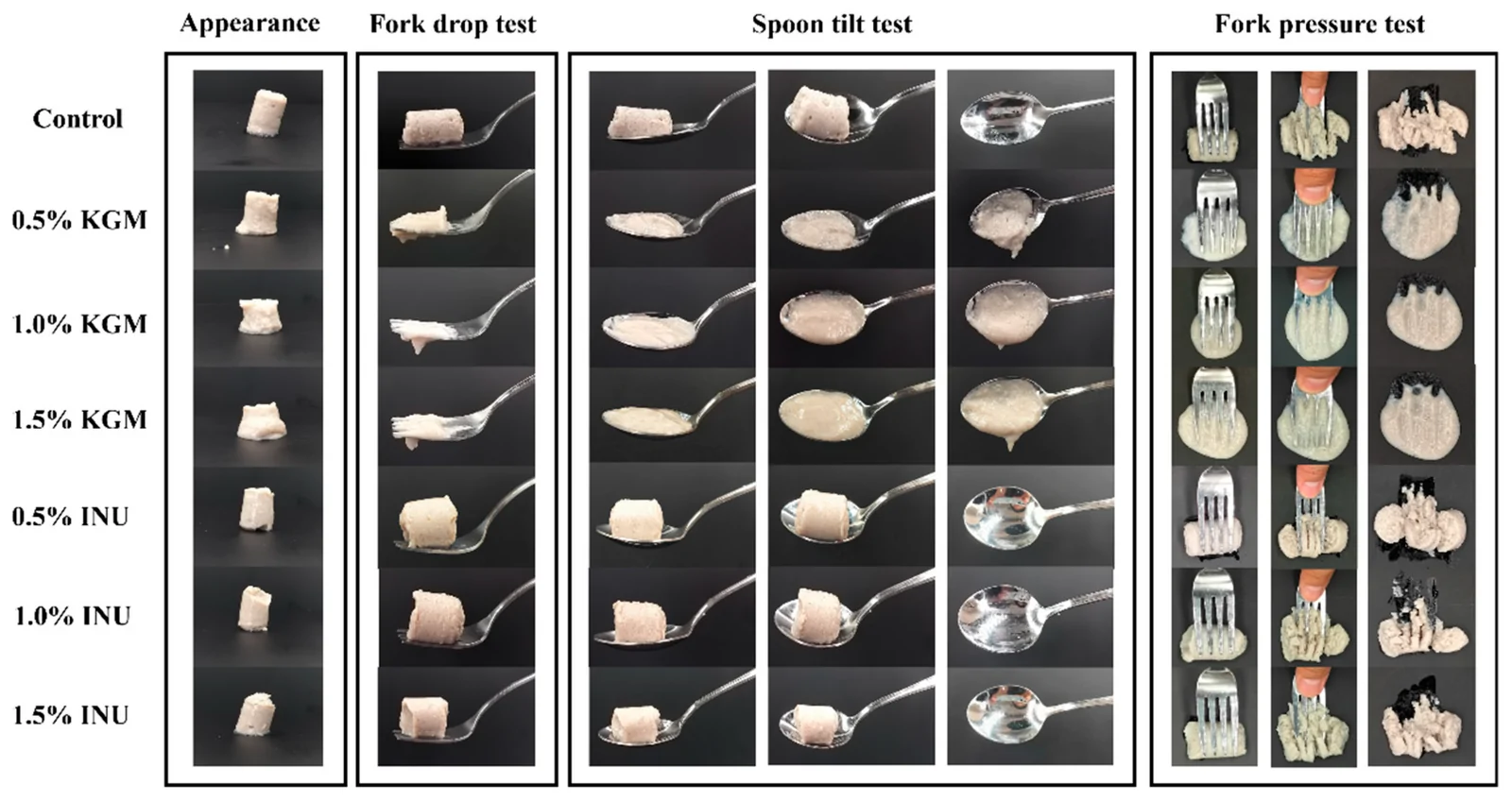
IDDSI evaluation of different SC-SC composite gels with different additions of KGM and INU. Note: KGM: konjac glucomannan; INU: inulin.

**Figure 3:**
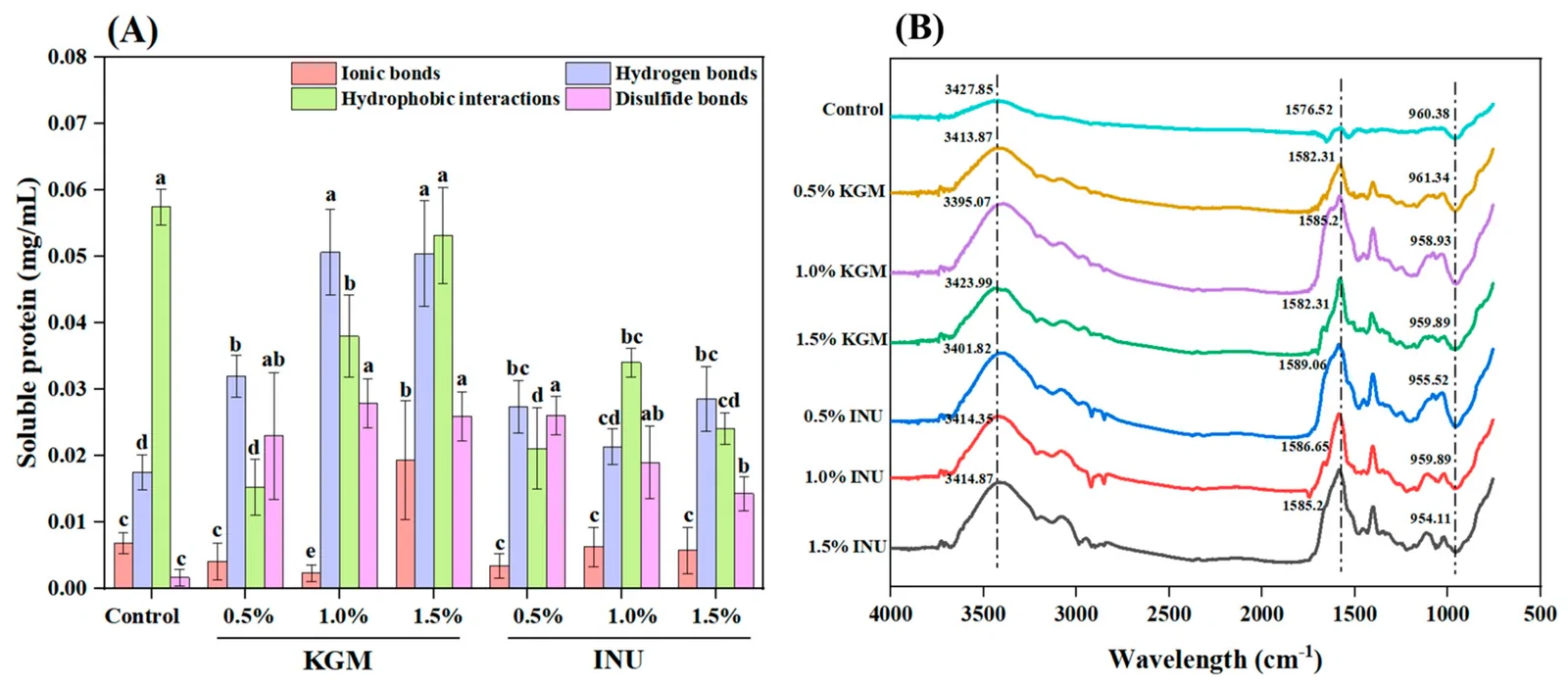
Intermolecular force (**A**) and FTIR spectra (**B**) of SC-SC composite gel with different addition of KGM and INU. Note: Different lowercase letters (a–e) within the same column indicate significant differences between groups (*p* < 0.05). KGM: konjac glucomannan; INU: inulin.

**Figure 4:**
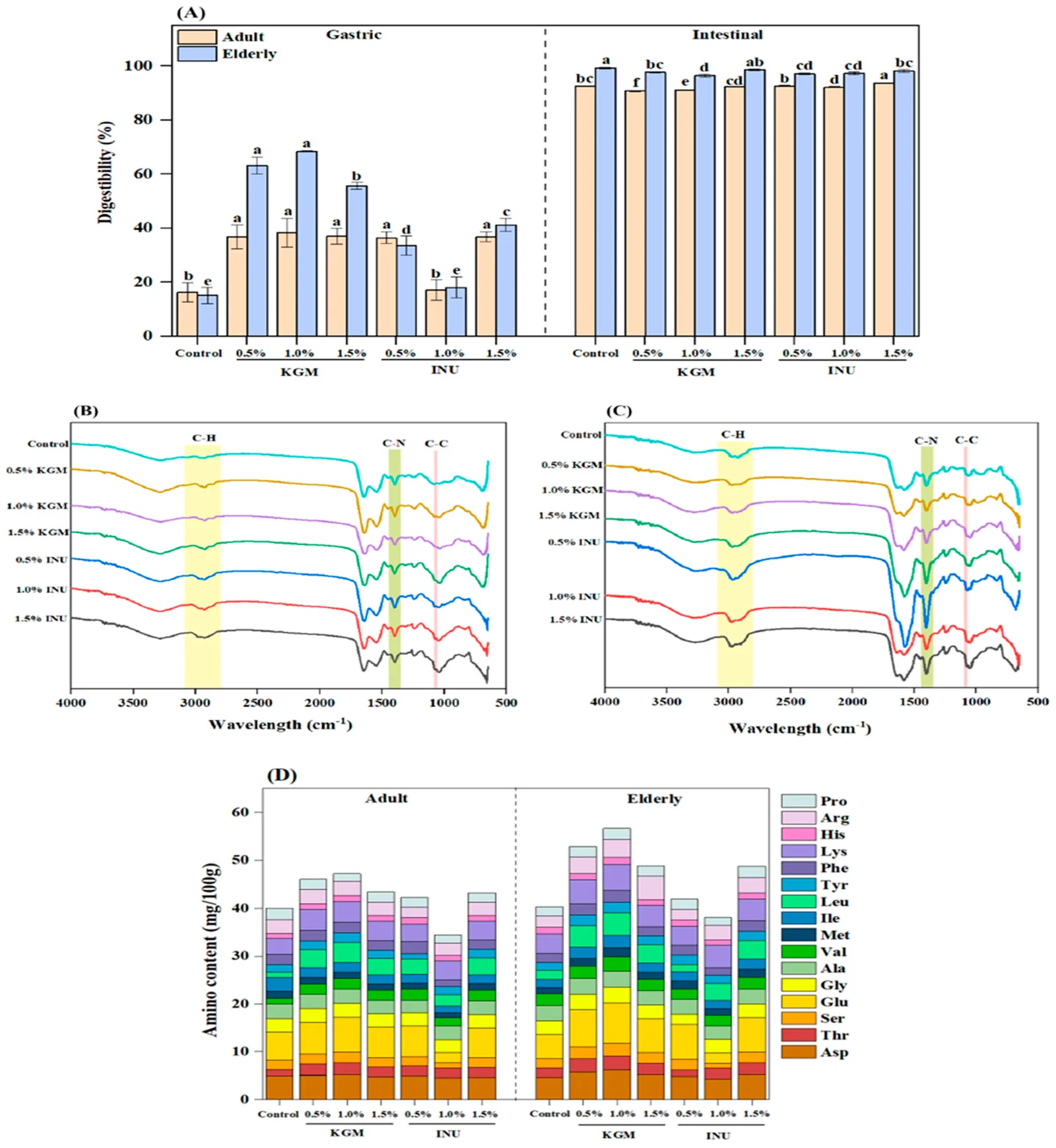
The digestibility (**A**), FTIR spectra under adult in vitro digestion model (**B**) and elderly in vitro digestion model (**C**), and amino acid contents (**D**) of different SC-SC composite gels under adult and elderly in vitro digestion models. Note: Digestibility values marked with different lowercase letters (a–f) indicate significant differences between groups (*p* < 0.05). KGM: konjac glucomannan; INU: inulin.

**Figure 5:**
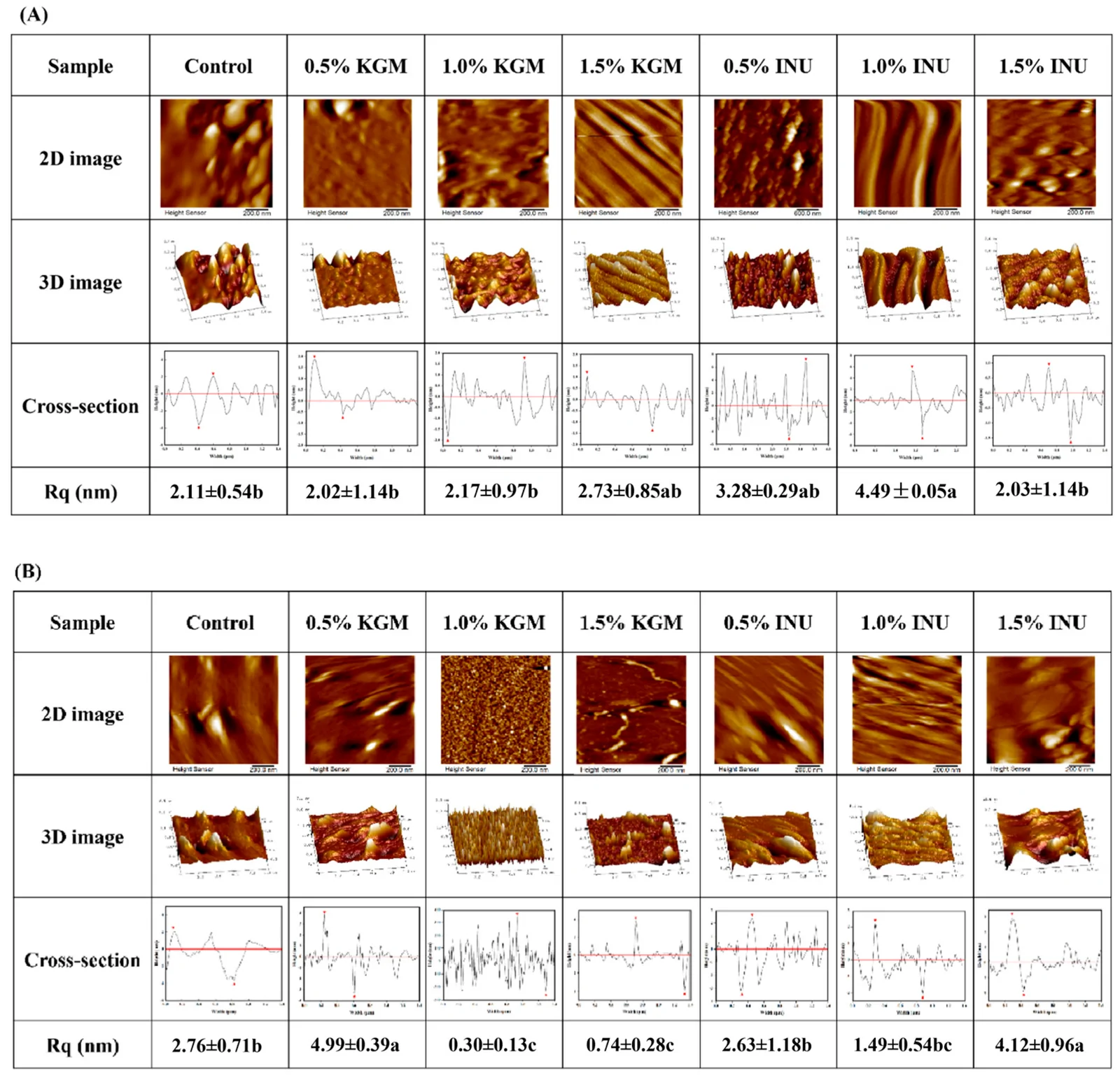
Atomic force microscopy (AFM) images of digestive products from different SC-SC composite gels under adult (**A**) and elderly (**B**) in vitro digestion models. Note: Rq values marked with different lowercase letters (a–c) indicate significant differences between groups (*p* < 0.05). KGM: konjac glucomannan; INU: inulin.

**Figure 6:**
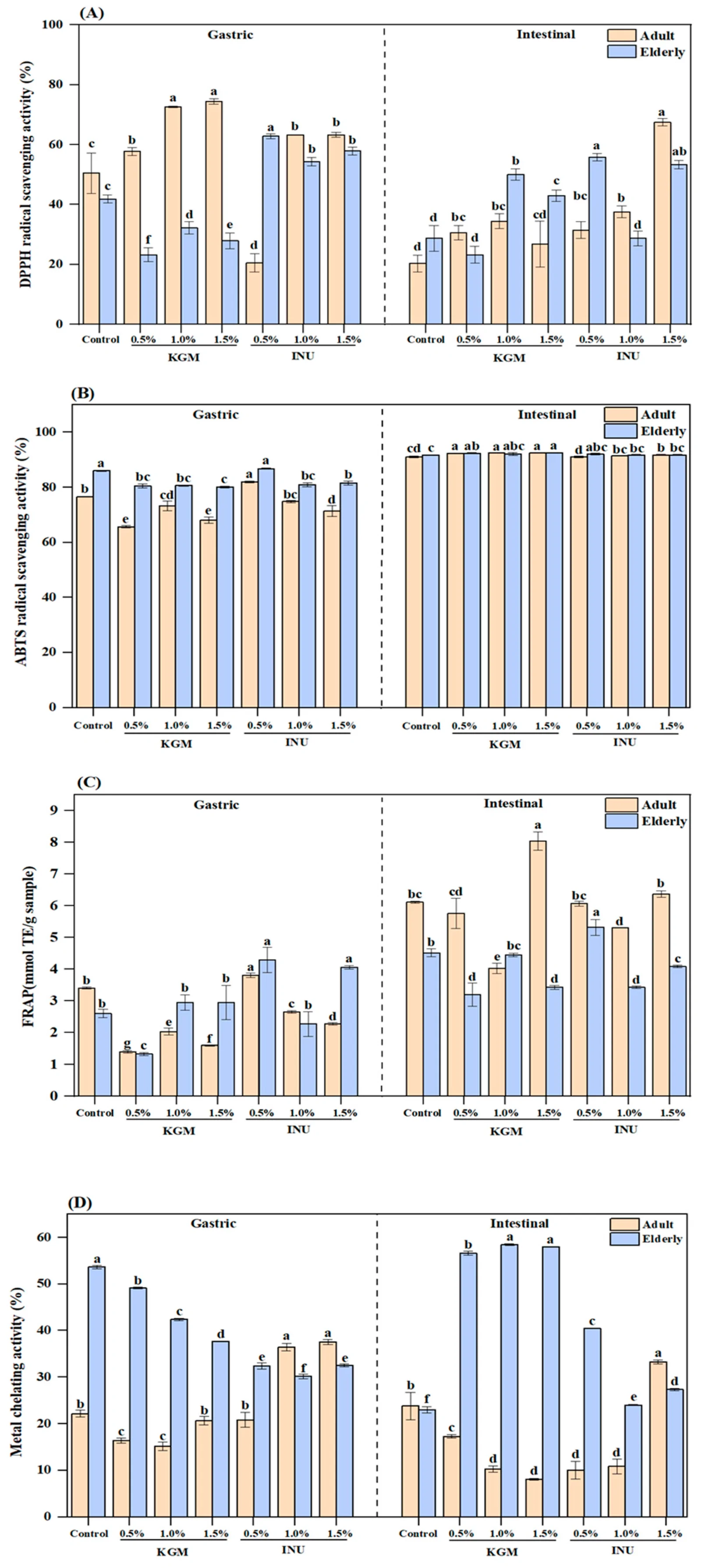
DPPH free radical scavenging activity (**A**), ABTS free radical scavenging activity (**B**), iron reduction ability (**C**) and metal chelating ability (**D**) of digestive products from different SC-SC composite gels under adult and elderly in vitro digestion models. Note: All data are presented as mean ± standard deviation (SD). Different lowercase letters (a–g) indicate significant differences between groups (*p* < 0.05); KGM: konjac glucomannan; INU: inulin.

**Table 1:** Textural properties of SC-SC composite gels with different additions of KGM and INU.

Sample		Hardness (N)	Cohesiveness	Springiness	Chewiness (mJ)	Adhesiveness (mJ)
Control		0.333 ± 0.047 a	0.493 ± 0.087 b	2.867 ± 0.25 ab	0.453 ± 0.103 b	0.210 ± 0.029 bc
KGM	0.5%	0.073 ± 0.018 b	0.700 ± 0.037 a	—	0.900 ± 0.042 a	0.343 ± 0.040 a
	1.0%	0.036 ± 0.004 b	0.777 ± 0.039 a	—	0.873 ± 0.053 a	0.273 ± 0.045 ab
	1.5%	0.052 ± 0.028 b	0.690 ± 0.054 a	—	0.737 ± 0.065 a	0.177 ± 0.050 cde
INU	0.5%	0.331 ± 0.031 a	0.483 ± 0.049 b	3.220 ± 0.326 a	0.513 ± 0.085 b	0.183 ± 0.054 cd
	1.0%	0.282 ± 0.021 a	0.513 ± 0.020 b	2.333 ± 0.307 b	0.330 ± 0.041 b	0.103 ± 0.012 de
	1.5%	0.320 ± 0.030 a	0.410 ± 0.090 b	2.707 ± 0.300 ab	0.373 ± 0.152 b	0.093 ± 0.009 e

Note: KGM: konjac glucomannan; INU: inulin. The values are the mean ± standard deviation. Lowercase letters indicate significant differences between groups (*p* < 0.05). “—” indicates that springiness was not detectable, because the gels were too soft to recover their original shape after compression.

## Data Availability

The original contributions presented in the study are included in the article/Supplementary Material, further inquiries can be directed to the corresponding authors.

## Supplementary Materials

The following supporting information can be downloaded at: https://www.mdpi.com/article/10.3390/foods15193471/s1, Table S1: Formulations of different SC-SC composite gels; Table S2: In vitro digestion solution and condition of adult and elderly digestive model.
