# Supplementary material for: Effects of Konjac Glucomannan and Inulin on the Gel Properties, Swallowing Characteristics, and In Vitro Digestion of Heat-Induced Silver Carp–Sea Cucumber Composite Gels for Dysphagia
Source: Foods. 2026 Sep 28;15(19):3471. doi: 10.3390/foods15193471 (PMC13665461; doi:10.3390/foods15193471)
Supplement: Supplementary file 1 [file foods-15-03471-s001.zip › foods-4548359-supplementary.pdf]

Table S1 Formulations of different SC-SC composite gels

Samples		Sea cucumber-fish paste (%)	Hydrophilic (%)		NaCl (%)
			KGM	Inulin	
Control		99	0	0	1
KGM	0.5%	98.5	0.5	0	1
	1.0%	98	1.0	0	1
	1.5%	97.5	1.5	0	1
INU	0.5%	98.5	0	0.5	1
	1.0%	98	0	1.0	1
	1.5%	97.5	0	1.5	1

Note. KGM: konjac glucomannan; INU: inulin; % is based w/w.

Table S2 *In vitro* digestion solution and condition of adult and elderly digestive model

Constituent	Adult						Elderly					
	SSF		SGF		SIF		SSF		SGF		SIF	
	mol/L	mL	mol/L	mL	mol/L	mL	mol/L	mL	mol/L	mL	mol/L	mL
KCl	0.5	15.1	0.5	6.9	0.5	6.8	0.5	15.1	0.5	6.9	0.5	6.8
KH_2PO_4	0.5	3.7	0.5	0.9	0.5	0.8	0.5	3.7	0.5	0.9	0.5	0.8
$NaHCO_3$	1	6.8	1	12.5	1	42.5	1	6.8	1	12.5	1	42.5
NaCl	——	——	2	11.8	2	9.6	——	——	2	11.8	2	9.6
$MgCl_2\cdot 6(H_2O)$	0.15	0.5	0.15	0.4	0.15	1.1	0.15	0.5	0.15	0.4	0.15	1.1
$(NH_4)_2CO_3$	0.5	0.06	0.5	0.5	——	——	0.5	0.06	0.5	0.5	——	——
pH	7.0		3.0		7.0		7.0		6.0		7.0	
Time	5 s		2 h		3 h		2 min		2 h		4 h	
Enzyme	α-amylase		porcine pepsin (2000 U/mL)		porcine trypsin (100 U/mL)		α-amylase		porcine pepsin (1500 U/mL)		porcine trypsin (50 U/mL)	
Rotate speed	200 rpm						60 rpm					
Temperature	37℃						37℃					
